# Metabolite and Proteomic Profiling of Serum Reveals the Differences in Molecular Immunity between Min and Large White Pig Breeds

**DOI:** 10.3390/ijms24065924

**Published:** 2023-03-21

**Authors:** Liyu Yang, Xin Liu, Xiaoyu Huang, Longchao Zhang, Hua Yan, Xinhua Hou, Lixian Wang, Ligang Wang

**Affiliations:** 1 Key Laboratory of Farm Animal Genetic Resources and Germplasm Innovation of Ministry of Agriculture of China, Institute of Animal Science, Chinese Academy of Agricultural Sciences, Beijing 100193, China; 2 College of Animal Sciences, Shanxi Agricultural University, Taigu 030800, China

**Keywords:** Min pigs, Large White pigs, metabolomic, proteomics, biomarker

## Abstract

Pig diseases seriously threaten the health of pigs and the benefits of pig production. Previous research has indicated that Chinese native pigs, such as the Min (M) pig, has a better disease resistance ability than Large White (LW) pigs. However, the molecular mechanism of this resistance is still unclear. In our study, we used serum untargeted metabolomics and proteomics, interrogated to characterize differences in the molecular immunities between six resistant and six susceptible pigs raised in the same environment. A total of 62 metabolites were identified as being significantly exhibited in M and LW pigs. Ensemble feature selection (EFS) machine learning methods were used to predict biomarkers of metabolites and proteins, and the top 30 were selected and retained. Weighted gene co-expression network analysis (WGCNA) confirmed that four key metabolites, PC (18:1 (11 Z)/20:0), PC (14:0/P-18: 0), PC (18:3 (6 Z, 9 Z, 12 Z)/16:0), and PC (16:1 (9 Z)/22:2 (13 Z, 16 Z)), were significantly associated with phenotypes, such as cytokines, and different pig breeds. Correlation network analysis showed that 15 proteins were significantly correlated with the expression of both cytokines and unsaturated fatty acid metabolites. Quantitative trait locus (QTL) co-location analysis results showed that 13 of 15 proteins co-localized with immune or polyunsaturated fatty acid (PUFA)-related QTL. Moreover, seven of them co-localized with both immune and PUFA QTLs, including proteasome 20S subunit beta 8 (PSMB8), mannose binding lectin 1 (MBL1), and interleukin-1 receptor accessory protein (IL1RAP). These proteins may play important roles in regulating the production or metabolism of unsaturated fatty acids and immune factors. Most of the proteins could be validated with parallel reaction monitoring, which suggests that these proteins may play an essential role in producing or regulating unsaturated fatty acids and immune factors to cope with the adaptive immunity of different pig breeds. Our study provides a basis for further clarifying the disease resistance mechanism of pigs.

## 1. Introduction

Pork provides humans around the world with about half of their animal protein resources. Modern pig production has a certain degree of antagonism between production traits and resistance traits, which show a negative genetic correlation. The pursuit of high-yield targets often results in reduced disease resistance [1]. At the same time, pig diseases, especially viral infectious diseases, seriously threaten the health of pigs and the benefits of pig production. The Min pig is an excellent local breed in China that has high immunity and strong disease resistance. Studies have shown that the number of red blood cells, the absolute value of neutrophils, and the percentages of T helper cells (CD4+T lymphocytes) and cytotoxic T cells (CD8+T lymphocytes) in Min pigs were significantly higher than in Large White pigs (*p* ≤ 0.01) [2,3]. In addition, the SLA class I gene has a stronger polymorphism in Min pigs [2]. When disease struck a farm, the growth or reproductive performance of Large White pigs was severely affected while that of the Min pigs in the same enclosure remained normal [4]. It would be of benefit to understand the molecular and genetic mechanisms of the immune system in different pig breeds.

Metabolites are effectively the end products of complex interactions occurring between structures inside the cell (the genome) and events, exposures, or phenomena occurring outside the cell or organism (the environment). As a result, metabolomics can enable researchers to obtain a sensitive and more complete description of the phenotype [5]. This metabolic readout of the phenotype is often called the “Metabotype” [6]. Thus, metabolomics is widely used to diagnose and prevent animal disease [7]. Proteins are the primary executors of cell function, and abnormal protein expression or modification in cells plays a vital role in the occurrence and development of diseases. Proteomics has become a critical technique for studying intracellular protein composition, activity, and interaction at the protein level. The porcine immune system has been investigated by profiling the proteomics for disease resistance research in pigs [8,9,10]. Blood combines metabolic and transcriptional variation and carries molecular signatures of system-wide processes, which makes blood widely used for integrative biomedical research [5,7,11,12,13,14]. However, few reports describe an integrated analysis of proteins and metabolites associated with Chinese domestic and foreign pig serum under common rearing conditions.

This study aims to comprehensively describe the complex interactions between different omics data using the combined effects of metabolomics and proteomics. To this end, we analyzed metabolomics and proteomics measurements from 12 individual Min (M) pigs and Large White (LW) pigs to explore the molecular mechanisms affecting the different pig breeds’ immune capacities. The results could provide insight into understanding differences in resistance between different breeds of pig and may be helpful for highly disease-resistant pig production.

## 2. Results

### 2.1. Metabolic Profiling of Serum Samples from M and LW Pigs

**Figure 1:**
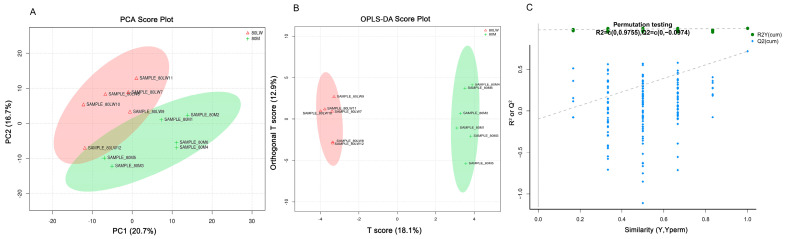
Principal component analysis (PCA). (**A**) PCA score plot of the serum of Large White (LW) and Min (M) pig metabolites; (**B**) Orthogonal partial least squares discrimination analysis (OPLS-DA) score plot; (**C**) The 200-response sorting permutation test plot in the OPLS-DA mode.

After data preprocessing and metabolite identification, 596 metabolites were extracted from the raw data acquired in positive and negative ion modes. We utilized a multivariate statistical analysis to explore the differences in metabolites between M and LW pigs. An unsupervised principal component analysis (PCA) analysis was performed as the first step in the separation procedure to visualize the clusters and decrease data dimension (Figure 1A). The distribution of two pig serum samples and the PCA shows a distinction between M and LW pigs.

Furthermore, we performed orthogonal partial least squares discrimination analysis (OPLS-DA) to further differentiate the metabolite features and to screen potential marker metabolites. In the OPLS-DA score plot, there are clear differences between the LW and M pigs (Figure 1B). In this study, the parameters R2Y = 0.994 and Q2 = 0.718 and this result indicated that the OPLS-DA models were not over-fitted and had a satisfactory interpretative and predictive ability, and so the data can be reliably used for further analysis (Figure 1C).

**Figure 2:**
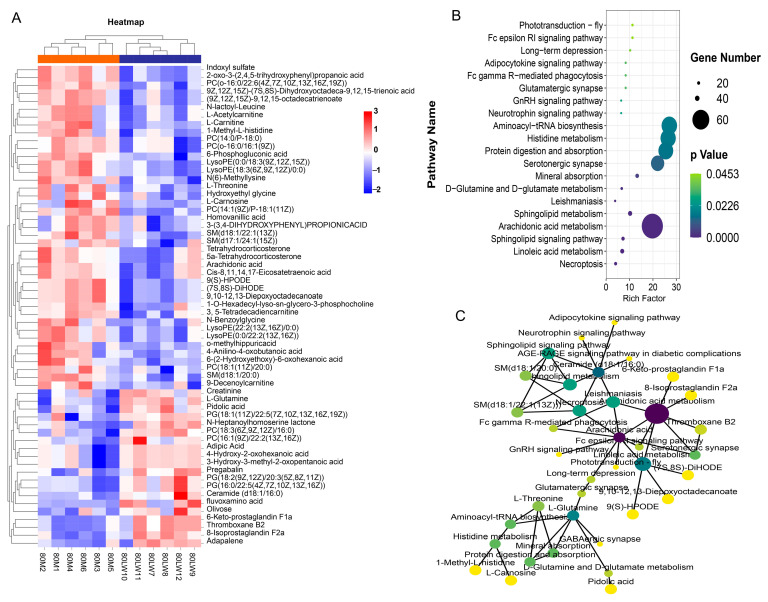
Quantitative metabolomics of LW and M pig serum samples. (**A**) Hierarchical clustering based on differential metabolites in the serum of LW and M pigs. The differentially expressed metabolites (DEMs) were identified with a VIP > 1 and *p*-value ≤ 0.05. Significantly altered metabolites are highlighted in red (increased) and blue (decreased); (**B**) Kyoto encyclopedia of genes and genomes (KEGG) pathway enrichment analysis of DEMs; (**C**) KEGG network diagram. The number of metabolites enriched into the pathway determines the node size, and the *p*-value determines the node color.

Based on pairwise comparisons of LW vs. M groups, 61 metabolites exhibited statistically significant differences at *p*-values ≤ 0.05 and a variable importance projection (VIP) value > 1. Hierarchical clustering analysis of these identified differentially expressed metabolites (DEMs) between the groups indicated two major express trajectories (Figure 2A) (Supplementary Table S1). In trajectory I, concentrations of 8-isoprostaglandin F2a, thromboxane B2, PC (16:19(9Z)/22:2(13Z,16Z)), 3-Hydroxy-3-methyl-2-oxopentanoic acid, adipic acid, 4-hydroxy-2-oxohexanoic acid, fluvoxamine acid, and pregabalin ceramide (d18:1/16:0) were upregulated in LW. In trajectory II, M pigs had 42 DEMs with higher expression than in LW, including L-glutamine, L-threonine, o-methyl hippuric acid, arachidonic acid, and creatinine.

A pathway enrichment analysis of differential metabolites showed that most metabolic pathways were involved in immune response or regulation, such as linoleic acid metabolism, the Fc epsilon RI signaling pathway, Fc gamma R-mediated phagocytosis, and arachidonic acid metabolism (Figure 2B). The kyoto encyclopedia of genes and genomes (KEGG) network diagram showed that arachidonic acid and sphingolipid metabolism, along with the sphingolipid signaling pathway and necroptosis pathway, were the core regions residing in all pathways (Figure 2C).

### 2.2. Identification of High-Confidence Biomarkers to Predict Immune Molecular Differences between Different Pig Breeds

**Figure 3:**
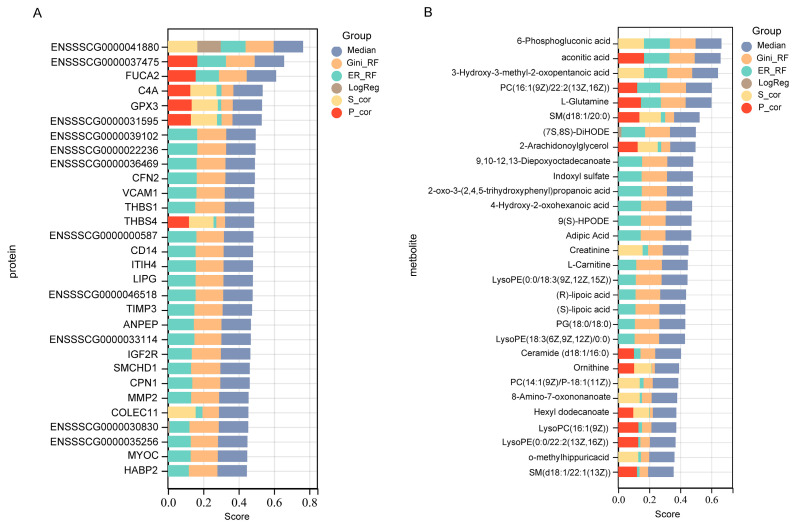
Identification of high-confidence biomarkers for predicting adaptive immune differences between pig breeds. (**A**) Top 30 ensemble feature selection (EFS) proteins (LW versus M); (**B**) Top 30 EFS metabolites (LW versus M).

In this study, one of our most important goals was the identification of high confidence biomarkers to predict immune molecular differences between different pig breeds. To rank biomarkers, we took an ensemble feature selection (EFS) approach, which applied multiple feature selection algorithms then aggregated and ranked the results. The top EFS protein (Figure 3A) were ENSSSCG00000037475, alpha-L-fucosidase 2 (FUCA2), complement C4A (C4A), glutathione peroxidase 3 (GPX3), vascular cell adhesion molecule 1 (VCAM1), and CD14 molecule (CD14). The top-ranked EFS metabolites included 6-phosphogluconic acid, PC(16:1(9z)/22:2(13z,16z)), 9,10-12,13-diepoxyoctadecanoate, and 9(S)-HPODE (Figure 3B). The EFS approach ensures that the top-ranked biomarkers are not correlated to one another and, therefore, could be used in combination for the enhanced prediction of immune differences in different pig breeds [15].

### 2.3. WGCNA Analysis to Identify Metabolite Modules Significantly Associated with Cytokines

**Figure 4:**
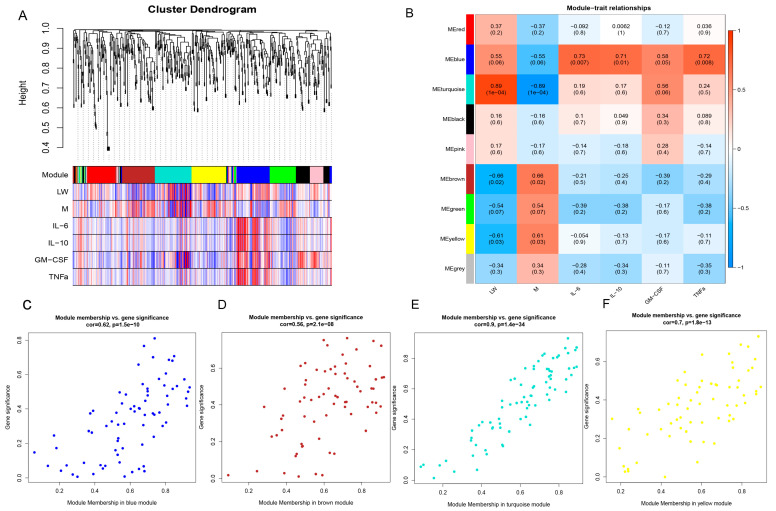
Weighted gene correlation network analysis (WGCNA) of all metabolites identified from different pig breeds. (**A**) Module clustering tree; (**B**) Phenotypic and modular correlation diagrams; (**C**–**F**) A scatterplot of gene significance (GS) vs. module membership (MM) in the four modules that showed the closest relationship with phenotypes.

In this study, we aimed to determine the relationship between serum metabolites and cytokine production in different pig breeds. We constructed a one-step metabolite-cytokine co-expression matrix to obtain nine modules. The blue module had the highest positive correlation with four cytokines (Figure 4A,B). It is worth noting that four cytokines, including cytokines interleukin 10 (IL10), cytokines interleukin 6 (IL6), tumor necrosis factor alpha (TNFα), and granulocyte-macrophage colony stimulating factor (GM-CSF), were discovered by our research group in previous studies to be differentially expressed in M pigs and LW pigs. The blue, turquoise, brown, and yellow modules also showed significant positive or negative correlations with different pig breeds. We identified highly significant metabolites with a high module membership in the above four modules using gene significance (GS) and module membership (MM) measures. Regarding the four modules significantly linked to different pig breeds and cytokines, each showed a significantly high correlation between GS and MM (Figure 4C–F). Our results identify metabolite modules that may directly affect cytokine expression, and these metabolites may contribute to immune differences in different pig breeds. In addition, KEGG and protein–protein interaction networks (PPI) interaction network analysis were also conducted to understand the complete picture of the metabolite functions in the four modules significantly related to immunophenotype (Supplementary Figures S1 and S2).

**Figure 5:**
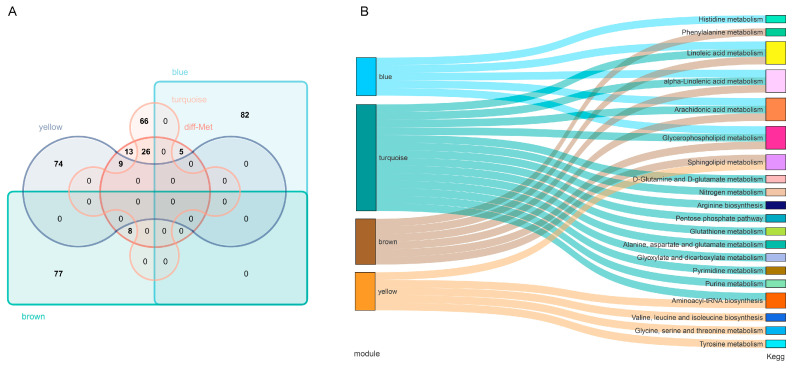
KEGG pathway enrichment analysis of four module metabolites. (**A**) Venn diagram of differential metabolites within the four modules; (**B**) KEGG enrichment analysis of four modular metabolites.

To better understand the function of the relevant metabolites in these four modules, we first identified the differential metabolites within these modules and performed KEGG analysis. There were 5, 26, 8, and 9 metabolites differentially expressed in the blue, turquoise, brown, and yellow modules, respectively (Figure 5A). KEGG enrichment results showed that the metabolites in blue, turquoise, brown, and yellow modules were mainly enriched in five pathways, namely linoleic acid, alpha-linolenic acid, arachidonic acid, glycerophospholipid acid, and sphingolipid metabolism pathways (Figure 5B).

**Figure 6:**
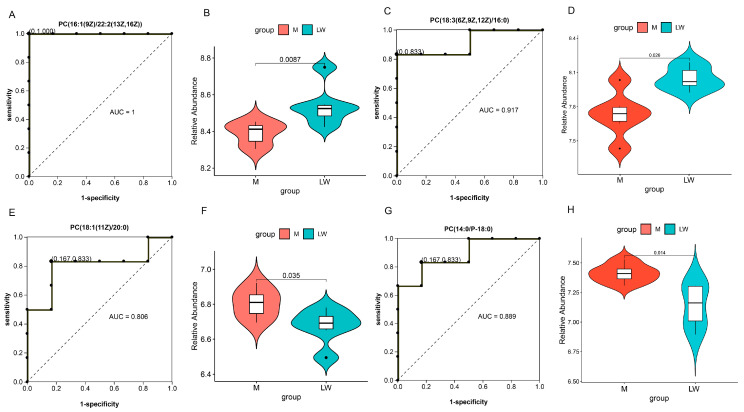
Receiver operating characteristic (ROC) curves and expression abundance of critical metabolites. (**A**,**C**,**E**,**G**) ROC curve for 4 key metabolites and (**B**,**D**,**F**,**H**) expression abundance of four metabolites in the serum of M pigs and LW pigs; red represents M pig, and blue represents LW pig.

The top KEGG enriched terms in these modules were mainly involved in fatty acid-related pathways. Interestingly, four metabolites belonging to phosphatidylcholine or lecithin, named PC (18:1(11Z)/20:0), PC (14:0/P-18:0), PC (18:3(6Z,9Z,12Z)/16:0), and PC (16:1(9Z)/22:2(13Z,16Z)), and were enriched in these four same pathways and deserve attention (Table 1). We analyzed the expression abundance (Figure 6B,D,F,H) of these four hub metabolites and plotted the receiver operating characteristic (ROC) curve (Figure 6A,C,E,G). The area under the curve (AUC) values of the four hub metabolites were all above 0.8, with the values of PC (16:1(9Z)/22:2(13Z,16Z)) reaching 1 (Figure 6). Therefore, we think that these four hub metabolites have high sensitivity and specificity for distinguishing differences in immunity between the two pig breeds.

### 2.4. Metadata Association Analysis of Biomarkers

**Figure 7:**
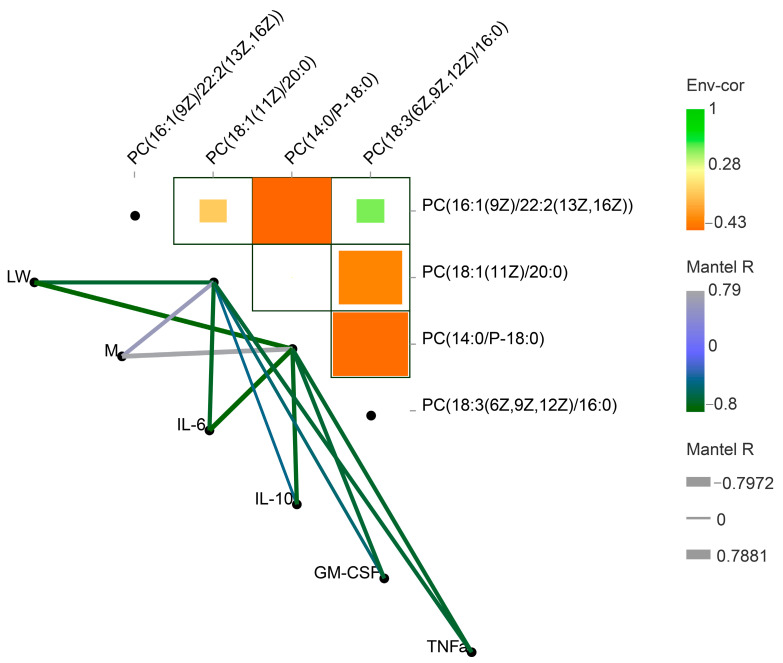
Metadata assessment of major biomarkers. The green line indicates a positive correlation, and the grey lines indicate a negative correlation. The thickness or thinness of the lines indicates strong and weak correlations between metabolites and phenotypes, respectively. *p*-values ≤ 0.05 were considered significant; *p*-values ≤ 0.01 were considered extremely significant. R2 ≥ |±0.5|, represents strong correlation.

Confounding factors have an important impact on the accuracy of biomarker analysis [16]. To investigate this issue, we performed a metadata-wide assessment for every multi-omic feature detected to investigate the confounding factors (Figure 7). The results showed that biomarkers (up and down-regulated) are predominantly associated with four cytokines and the breed of pig.

### 2.5. Correlation Network Analysis to Screen Key Proteins Affecting Cytokine and Metabolite Expression

**Figure 8:**
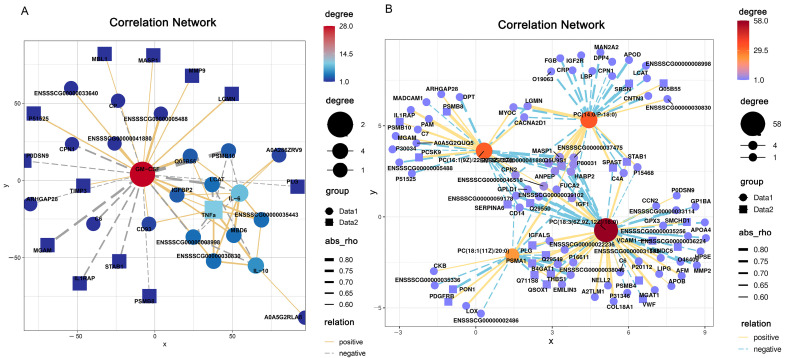
Correlation interaction network analysis based on significantly different proteins and metabolites. (**A**) Protein-cytokine interaction network analysis; (**B**) Protein-metabolite interaction network analysis. The yellow dashed line indicates a positive correlation, and the grey (**A**) and blue (**B**) dashed lines indicate a negative correlation. The thickness or thinness of the dashed lines indicates strong and weak correlations, respectively. *p*-values ≤ 0.05 were considered significant; *p*-values ≤ 0.01 were considered extremely significant. R2 ≥ |±0.5| represents strong correlation.

In order to know whether proteins could regulate the induction of cytokines, protein-cytokine interaction network analysis was employed based on differentially expressed proteins (DEPs) and cytokines. A total of 540 correlations were found between 135 proteins and 4 cytokines. Among the 540 correlations, 54 were significant (*p*-value ≤ 0.05, including 30 proteins), of which 33 were negative and 21 were positive, R^2^ ≥ |±0.5| (Figure 8A). In order to investigate whether proteins regulate cytokines by affecting the expression of metabolites, we performed a correlation analysis on 135 proteins and 4 metabolites. A total of 540 correlations were found between 135 DEPs and 4 metabolites. Among the 540 correlations, 145 were significant (*p*-value ≤ 0.05, including 97 proteins), of which 88 were negative and 57 were positive, R2 ≥ |±0.5| (Figure 8B). A KEGG enrichment analysis was used to explore the potential biological functions of the proteins in the correlation network. The 30 proteins from the protein-cytokine network were mainly enriched in complement and coagulation cascades, Staphylococcus aureus infection, and antigen processing and presentation (Supplementary Figure S3A). The 97 proteins from the protein-cytokine network were primarily involved in cell adhesion, prion disease, staphylococcus aureus infection, the NF-kappa B signaling pathway, and the complement and coagulation cascades pathways (Supplementary Figure S3B). These results suggest that these proteins may play an important role in some diseases or immune responses.

### 2.6. Co-Location Analysis of Key DEPs and QTLs

**Figure 9:**
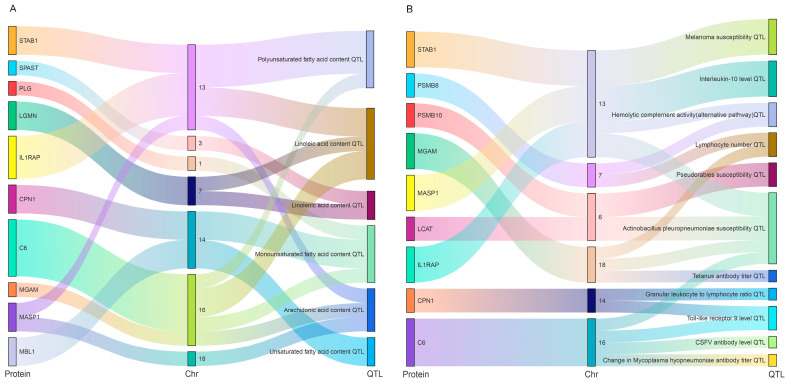
In-depth analysis of potential biological functions of 15 key proteins based on pig quantitative trait locus (QTL) data and protein co-localization analysis. (**A**) The intersection of 15 key proteins with polyunsaturated fatty acid-related QTL data; (**B**) The intersection of 15 key proteins with pig immunity-related QTL data.

Immune and polyunsaturated fatty acid-related traits from the pig quantitative trait loci database (QTLdb) were used for QTL co-location analysis of proteins significantly associated with cytokines and unsaturated fatty acid metabolites (30 and 97 proteins, respectively). We found that 19 of 30 DEPs co-localized with immune-(17 immune-related QTL) or PUFA-associated QTL (12 fatty acid-related QTL) traits (Figure 9). In addition, 53 of 97 proteins co-localized with immune- (42 immune-related QTL) or PUFA-associated QTL (45 fatty acid-related QTL) traits (Figure 9) (Supplementary Table S2). Interestingly, 15 proteins were found to be strongly correlated with 4 cytokines and 4 metabolites simultaneously in both groups (Supplementary Figure S4). In addition, 12 of 15 DEPs, such as MBL1, IL1RAP, and PSMB8, co-localized with immune- (10 immune-related QTL) or PUFA-associated QTL (9 fatty acid-related QTL) traits (Figure 9). Their expression levels were verified by parallel reaction monitoring (PRM). The results indicate that these proteins may play an important role in producing or regulating unsaturated fatty acids and immune factors to regulate the immunity of different pig breeds.

### 2.7. PRM Validation of Key DEPs

**Figure 10:**
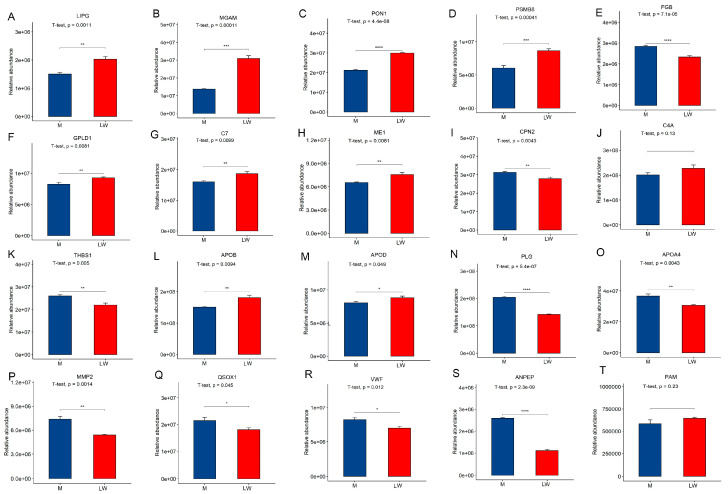
Quantification validation of proteins by parallel reaction monitoring (PRM). (A–T) Quantitative results of PRM for some differential proteins. *p*-value ≥ 0.05: no marks; *p*-value ≤ 0.05: *; *p*-value ≤ 0.01: **; *p*-value ≤ 0.001: ***; *p*-value ≤ 0.0001: ****.

We selected some critical candidate proteins from 112 DEPs, including MGAM, PSMB8, THBS1, and HABP1, and performed parallel reaction monitoring (PRM) for expression validation (Figure 10). The results are consistent with the data-independent acquisition (DIA) results, indicating the reliability and repeatability of the DIA-derived proteomics results in our study.

## 3. Discussion

The economic loss to pig production caused by disease accounts for about 12–15% of the total output value. Exploring the mechanisms of differential disease resistance in Chinese local and foreign pig breeds may help us to improve pig health and productivity. In-depth analysis of serum proteomics and metabolites using “omics” methods can help to identify biomarkers that contribute to differences in immunity levels in animals and study the molecular mechanisms of the pathogenesis, which could be applied to disease resistance breeding in the future. In our study, PCA analysis results showed that M pigs and LW pigs could be distinguished, indicating significant differences in metabolites between them. The OPLS-DA score map is a supervised pattern recognition method, and the results further suggest that the serum metabolites of M pigs are significantly different from those of LW pigs, and this warrants further investigation.

Metabolic reprogramming plays an essential role in the development and activation of immune cells in response to pathogen challenges or environmental changes [17]. Emerging evidence suggests a key role of metabolic regulation in cytokine release [18,19,20]. Research shows that lipopolysaccharide (LPS) induces the release of succinate in macrophages, which further induces IL-1β production in an autocrine manner [21]. Dietary supplementation with alpha-ketoglutaric acid induces the production of IL10, which inhibits chronic inflammation and extends life span [22]. Extensive studies have proved that metabolites and pro-inflammatory cytokines are inextricably linked. In our previous study, cytokines IL6, IL10, TNFα, and GM-CSF were highly expressed in LW pigs. Thus, we used all metabolites supplemented with cytokines and pig breeds as phenotypes to construct WGCNA and to further infer the potential relationship between metabolites and differences in immunity between different pig breeds. Among the nine modules, a total of four significant correlation modules were identified. The blue module metabolites were significantly correlated with four cytokines, indicating a special relationship and the importance of these metabolites in immunity. Combined with differential metabolite analysis, we identified the metabolite subsets responsible for the differences in pig breed immunity. A KEGG enrichment analysis of these differential metabolites showed that these metabolites were generally involved in lipid metabolism-related pathways. In particular, the metabolites PC(18:1(11Z)/20:0), PC(14:0/P-18:0), PC(18:3(6Z,9Z,12Z)/16:0), and PC(16:1(9Z)/22:2(13Z,16Z)) participate in linoleic acid (LA), alpha-linolenic acid (ALA), arachidonic acid (AA), and glycerophospholipid metabolism pathways, suggesting that these four key metabolites have potential predictive roles in distinguishing immunity differences between pig breeds.

Phosphatidylcholine (PC), also named lecithin, is a lipid class of fundamental biological importance, constituting the major structural component of cellular membranes and as a precursor that mediates essential fatty acid metabolism. Phospholipase A2 (PLA2) mediates the hydrolysis of phosphatidylcholine at the Sn -2 position on the phospholipid backbone and is involved in the metabolism of omega-3 and omega-6, also directly yielding a free AA molecule in one single step [23]. At present, there are few reports about lecithin being directly involved in disease or the immune response. Phosphatidylcholine is often used as an important precursor in the metabolism of lysophosphatidylcholine (Lyso PC), sphingomyelin (SM), and polyunsaturated fatty acids (PUFA). PCs are a major source of Lyso PC and play an essential role in regulating cellular lipid metabolism and homeostasis through the Lands cycle. Lyso PC binds to the G protein-coupled receptors (G2A), which activate MAPK, including ERK1/2, p38MAPK, JNK, and Toll-like receptors, thereby inducing oxidative stress, chemokine expression, and inflammation [24]. In the cis-Golgi apparatus, sphingomyelin synthase 1 (SMS1) catalyzes the transfer of a phosphorylcholine group from PC to ceramide to generate sphingomyelin (SM) [25]. SM is transported to the plasma membrane in vesicles and forms lipid rafts with cholesterol, participating in the development of atherosclerosis, cancer, and other diseases [26]. PC is also the main precursor molecule of arachidonic acid (AA) [27]. As a second messenger, AA is involved in signal transduction in the physiological processes of different diseases. AA can not only activate NADPH oxidase and induce oxidative stress, but can also be converted into prostaglandin D2 or prostaglandin E2 to form compounds with anti-inflammatory properties [28]. All in all, phosphatidylcholine, as an important precursor, plays an important role in different types of fatty acid metabolism.

**Figure 11:**
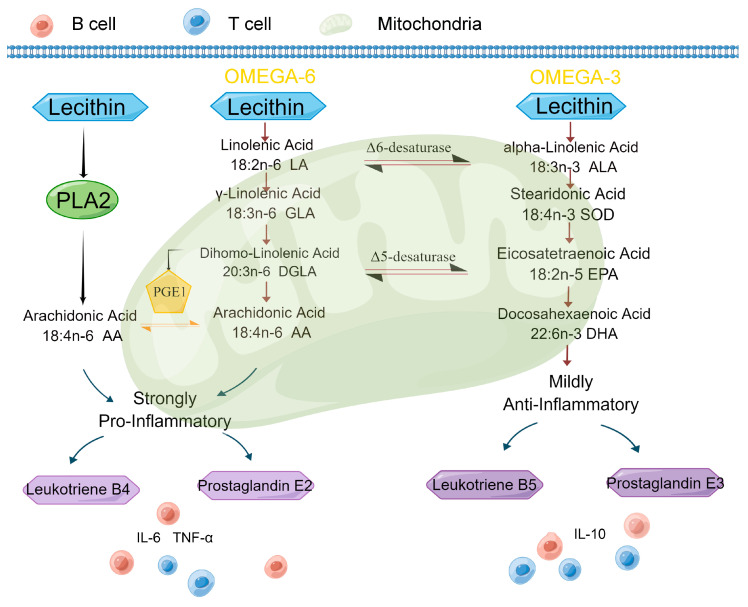
Phosphatidylcholine as a precursor in unsaturated fatty acid metabolism induces immune cell activation to participate in immunity tolerance.

Omega-3 and omega-6 regulate the differentiation of immune cells (B and T cells) [29]. Both cells require abundant nutrients to undergo expansion, proliferation, and differentiation upon immune challenge [30]. In our KEGG enrichment analysis of differential metabolites and WGCNA significance modules, multiple fatty acid metabolic pathways were identified, suggesting that fatty acid metabolism may be related to differences in immunity between different pig breeds. We found that phosphatidylcholine is strongly associated with the expression of cytokines and with the breed of pig. In fact, phosphatidylcholine acts as a precursor in the metabolism of different types of lipids, most importantly in the metabolism of polyunsaturated fatty acids (PUFAs). PUFAs are known as specialized pro-resolving lipid mediators (SPMs) and are mainly involved in the regression of inflammation and the promotion of adaptive immunity [31,32]. Arachidonic acid, linoleic acid, and α-linoleic acid belong to the omega-6 and omega-3 group of polyunsaturated fatty acids, respectively. Eicosanoids arising from arachidonic acid (omega-6) induce a pro-inflammatory response via 2-series and 4-series prostaglandin, inflammatory cytokine (IL6, TNFα), and leukotriene synthesis. ALA (omega-3) is the precursor of eicosapentaenoic acid (EPA) and docosahexaenoic acid (DHA) and so is converted into EPA and DHA [33]. EPA and DHA can partly inhibit aspects of inflammation, including leucocyte chemotaxis, cytokines (IL10), adhesion molecule expression, and leucocyte-endothelial adhesive interactions, and ultimately achieve inflammatory resolution (Figure 11). ARA, EPA, and DHA are converted into lipid mediators under the catalysis of lipoxygenase, inhibiting the inflammatory process [34]. Inflammatory resolution or extinction is an important active stage that is mediated by small molecules that are the products of omega-6 and omega-3 acid metabolism.

Targeted MS-based protein quantification, such as multiple reaction monitoring (MRM)-MS and parallel reaction monitoring (PRM)-MS, is the commonly used validation method in proteomics. In this study, we used PRM technology to quantify some proteins identified in the correlation network. PRM results confirmed that PSMB8 was upregulated in LW pigs. The PSMB8 gene encodes an essential subunit of a specialized immunoproteasome complex. The generated peptides have a higher affinity with major histocompatibility complex (MHC) I molecules and in turn enhanced antigenicity to CD8+ T cells [35,36]. Interleukin-1 receptor accessory protein (IL1RAP) is an innate immune mediator that regulates the activation of pro-inflammatory and mitogenic signaling pathways. Because of funding issues, we did not perform PRM analysis on all proteins screened in the correlation network. However, that does not mean they do not play an important role in molecular immunity. IL1RAP is involved in three signaling pathways that influence the expression of many IL-1 family cytokines (IL-1α, IL-1β, IL-33, IL-36β, and IL-36γ) in a variety of diseases [37]. MBL is a pattern-recognizing serum protein that participates in the innate immune system of mammals as an opsonin [38]. The antimicrobial function of MBL includes opsonization, neutralization, and complement activation [39]. MBL1 in pigs and cattle is considered as a candidate gene for mastitis resistance [40,41]. Our results suggest that differential expression of these proteins may regulate the secretion of cytokines by immune cells that are involved in the body’s innate and adaptive immune responses that maintain homeostasis. In general, under the same growing environment, the physiological state of M pigs was relatively stable and had stronger tolerance.

## 4. Materials and Methods

### 4.1. Subjects and Tissue Collection

We selected 12 piglets from 2 littermates of different pig breeds (Min pig (n = 6) and Large White (n = 6) from Changping Breeding Pig Farm, Institute of Animal Sciences, Chinese Academy of Agricultural Sciences). From each litter, piglets were selected based on their weight at 28 days (the average weight of M pigs was 8 ± 1.52 kg, and LW pigs was 10.50 ± 1.54 kg), avoiding underweight or overweight individuals. The piglets were raised for 35 days in the farrowing house and 45 days in the nursery house. The pigs were raised in the same environment and were fed the same diets. These diets consisted of 2270 kcal/kg net energy, 19.5% crude protein, 2.5% crude fat (the ingredient composition of diets added in Supplementary Table S3). The average daily feed intake during the feeding period was 520 g for M pigs and 650 g for LW pigs. The average weight of 80-day-old M and LW pigs was 25.2 ± 2.3 kg, and 33.4 ± 2.9 kg, respectively. Serum samples were collected at 80-day-old and stored at −80 °C until use.

### 4.2. LC-MS/MS for Analysis of Metabolomics

A Dionex ultimate 3000 ultra-high-performance liquid chromatographic (UPLC) system was used to perform the chromatographic separation. One microliter of the sample was injected into a Thermo Syncronis C18 column (1.7 μm, 2.1 mm × 100 mm) under a flow rate of 0.3 mL/min at 35 °C). The mobile phases consisted of 0.1% formic acid and 2 mmoL ammonium formate in water (mobile phase A) and acetonitrile (mobile phase B). The gradient elution conditions were performed as follows: 0−1 min, 95−40% A; 5−8 min, 40−0% A; 8−11 min, 0% A; 11−14 min, 0−40% A; 11−15 min, 40−95% A; and 15−18 min, 95% A. A random order was adopted for continuous analysis of samples to avoid the influence of instrument detection signal fluctuation.

### 4.3. Mass Spectrometry Conditions

Both negative and positive ionization modes were selected using the electrospray ionization (ESI) interface. The electrospray voltage was 2.8 kV, the sheath gas flow rate was 35 arb, the auxiliary gas flow rate was ten arb, and the capillary temperature was 320 °C. The resolution of the full scan was 70,000, and the scanning range was 70~1050 (*m*/*z*). For the second-level data dependence scan (Full MS/DD-MS), the resolution was 17,500, and the stepped NCE values were 20, 40, and 60 V.

### 4.4. Data Quality Control and Pre-Processing

Highly consistent data processing pipelines and analytical methods are used to improve the accuracy of metabolomics studies. TraceFinder 3.2.0 software was used to process the original data to obtain a data matrix, with information including retention time and peak intensity. A support vector regression method was used to correct filtered peaks. All detected ions were normalized based on the relative intensity between the area of the detected feature and the area of the QC samples (equation: area[feature]/area [QC]). In addition, variables with a relative standard deviation (RSD) of ≥30% for QC samples were removed. Log10 converted data were used to obtain the data matrix for subsequent analysis.

### 4.5. Identification of Differential Expressed Metabolites (DEMs) and Related Pathway

Differential metabolites were identified based on variable importance in projection (VIP) ≥ 1 (generated by the OPLS-DA model) and *p*-value ≤ 0.05. Differential metabolites were mapped into the Kyoto Encyclopedia of Genes and Genomes (KEGG) pathway database (http://www.kegg.jp/kegg/pathway.html, accessed on 8 November 2022) to analyze their metabolic pathways.

### 4.6. Construction of the Data-Independent Acquisition (DIA) Spectral Library and Library Searches

This was as described in our previous study [4].

### 4.7. Identification of High-Confidence Biomarkers Based on Machine Learning EFS

The ensemble feature selection (EFS) approach, which can reduce the biases of any individual feature selection method, was implemented in R using the EFS package [42]. The EFS approach combines MWU tests, logistic regression, Pearson and Spearman correlations, and two random forest algorithm implementations (forest and random forest) into a single, rankable score. The average score from both binary comparison analyses ultimately ranked the biomarkers.

### 4.8. Screening of Candidate Biomarkers

The differential metabolite data from the Large White and Min pigs were imported into GraphPad Prism 7.0 software to draw the receiver operating characteristic (ROC) curve. The ROC curve was plotted at each point with the true positive rate (TPR, sensitivity) as the ordinate and the false positive rate (FPR) as the abscissa, and then, the area under the curve (AUC) was calculated. An indicator biomarker group distinguishes between two groups, usually between 0.5 and 1.0, and the larger the area, the better the prediction. A metabolite with AUC > 0.7 is considered to have high predictive effect on disease and can be used as a potential biomarker for further study.

### 4.9. Weighted Gene Correlation Network Analysis (WGCNA) for the Determination of Critical Modules

The WGCNA R-package was used for co-expression network analysis. Screening modules are significantly related to pig breed phenotype. The Pearson method was used to calculate the correlation coefficients between genes. The adjacency matrix was transformed into the topological overlapping matrix (TOM), and hierarchical clustering was used to generate a hierarchical clustering tree of genes. Gene significance (GS) and modular significance (MS) were calculated to measure the importance of genes and pig breed and to analyze the significant association between modules and models.

### 4.10. Construction of the Protein–Protein Interaction Networks (PPI) Network

The metabolite data in the core module was uploaded to the search tool STRING (http://string-db.org, accessed on 11 November 2022) to search for interacting genes/proteins. The node and edge information exported as a .txt file and visualized using Cytoscape software (http://cytoscape.org/, version 3.7.2, accessed on 11 November 2022). The cytoHubba plugin was used to analyze degree scores, and the metabolites with the highest score were used to build the PPI sub-network.

### 4.11. KEGG Enrichment Analysis of Metabolomics Data

MetaboAnalyst (https://www.metaboanalyst.ca/, accessed on 11 November 2022) was used to perform KEGG enrichment analyses for differentially expressed metabolites (DEMs). KEGG pathways with corrected; *p*-values ≤ 0.05 were considered significantly enriched.

### 4.12. Correlation Analysis between the Proteins and Metabolites

OmicStudio tools (https://www.omicstudio.cn/tool, accessed on 14 November 2022) were used to calculate the correlation of continuous data between differentially expressed proteins and four metabolites based on Pearson correlation (*p*-value ≥ 0.05: no marks; *p*-value ≤ 0.05 represents significant correlation; *p*-value 0.05, representing extremely significant correlation). The yellow dashed line indicates a positive correlation, and the blue and gray dashed lines indicate a negative correlation. The judgment interval for strong or weak correlation is as follows: R2 ≥ |0.5|, represents strong correlation; R2 =|0.3~0.5|, represents moderate correlation; R2 =|0.1~0.3|, represents low correlation.

### 4.13. Differentially Expressed Proteins (DEPs) and QTL Co-Location Analysis

A total of 35,846 QTLs from 773 publications containing 693 phenotypic traits were collected in the current release of the Pig QTLdb (https://www.animalgenome.org/cgi-bin/QTLdb/SS/index, accessed on 16 November 2022). DEPs overlapped with QTLs in the pig QTLdb, and previous reports of the immune trait were used to screen the DEPs for the candidate genes associated with a pig’s adaptive immunity.

### 4.14. Parallel Reaction Monitoring (PRM) Analysis

The peptide digests of serum were separated and analyzed using Easy nLC 1200 and Q-Exactive HFX, respectively. The target protein data were imported into Skyline 4.1 software to obtain the chromatographic peak comparison for each peptide in different samples. The raw file was imported into the original DDA data by MaxQuant software v2.3.1.0 to screen the matching peptides with high ionic strength and fewer heteropeaks. Database retrieval parameters were as follows: missed cleavage was set to 0 and the reliability of the peptide was greater than 95%. Finally, the database search results were imported into Skyline 4.1 software to compare the selected candidate peptides.

### 4.15. Statistical Analysis

Principal component analysis (PCA) was performed using MetaboAnalyst 5.0 (https://www.metaboanalyst.ca/, accessed on 16 November 2022). Orthogonal projections to latent structures discrimination analysis (OPLS-DA) and variable importance in projection (VIP) values were generated using MetaboAnalyst R. Fisher’s exact test was applied to identify the significant KEGG pathways, *p*-value ≤ 0.05.

## 5. Conclusions

In conclusion, in this study, we screened four core metabolic pathways, four key metabolites, and 15 key proteins that affect molecular immunophenotypes (cytokines). Some metabolites, such as PC(18:1(11Z)/20:0), PC(14:0/P-18:0), PC(18:3(6Z,9Z,12Z)/16:0), and PC(16:1(9Z)/22:2(13Z,16Z)), may be critical biomarkers for immune responses and are expected to be helpful for future breeding of disease-resistant pigs. Fifteen key proteins, including PSMB8, MBL1, and IL1RAP, may play an important role in the production or metabolism of unsaturated fatty acids and immune factors.

## Figures and Tables

**Table 1:** Metabolites in the four core pathways.

Linoleic Acid Metabolism	Alpha-Linolenic Acid Metabolism	Arachidonic Acid Metabolism	Glycerophospholipid Metabolism
PC (16:1(9Z)/22:2(13Z,16Z))	PC (16:1(9Z)/22:2(13Z,16Z))	PC (16:1(9Z)/22:2(13Z,16Z))	PC (16:1(9Z)/22:2(13Z,16Z))
PC (18:3(6Z,9Z,12Z)/16:0)	PC (18:3(6Z,9Z,12Z)/16:0)	PC (18:3(6Z,9Z,12Z)/16:0)	PC (18:3(6Z,9Z,12Z)/16:0)
PC (14:0/P-18:0)	PC (14:0/P-18:0)	PC (14:0/P-18:0)	PC (14:0/P-18:0)
PC (18:1(11Z)/20:0)	PC (18:1(11Z)/20:0)	PC (18:1(11Z)/20:0)	PC (18:1(11Z)/20:0)
(7S,8S)-DiHODE		6-kKeto-prostaglandin F1a	
9,10-12,13-Diepoxyoctadecanoate		8-isoprostaglandin F2a	
		thromboxane B2	

## Data Availability

The datasets presented in this study can be found in online repositories. The names of the repositories and accession number(s) can be found in Section 4.

## Supplementary Materials

The following supporting information can be downloaded at: https://www.mdpi.com/article/10.3390/ijms24065924/s1.
