# Supplementary material for: Metabolite and Proteomic Profiling of Serum Reveals the Differences in Molecular Immunity between Min and Large White Pig Breeds
Source: Int J Mol Sci. 2023 Mar 21;24(6):5924. doi: 10.3390/ijms24065924 (PMC10056118; doi:10.3390/ijms24065924)
Supplement: Supplementary file 1 [file ijms-24-05924-s001.zip › Supplementary Figures.pdf]

Metabolite and Proteomic Profiling of Serum Reveals the Differences in Molecular Immunity between Min and Large White Pig Breeds

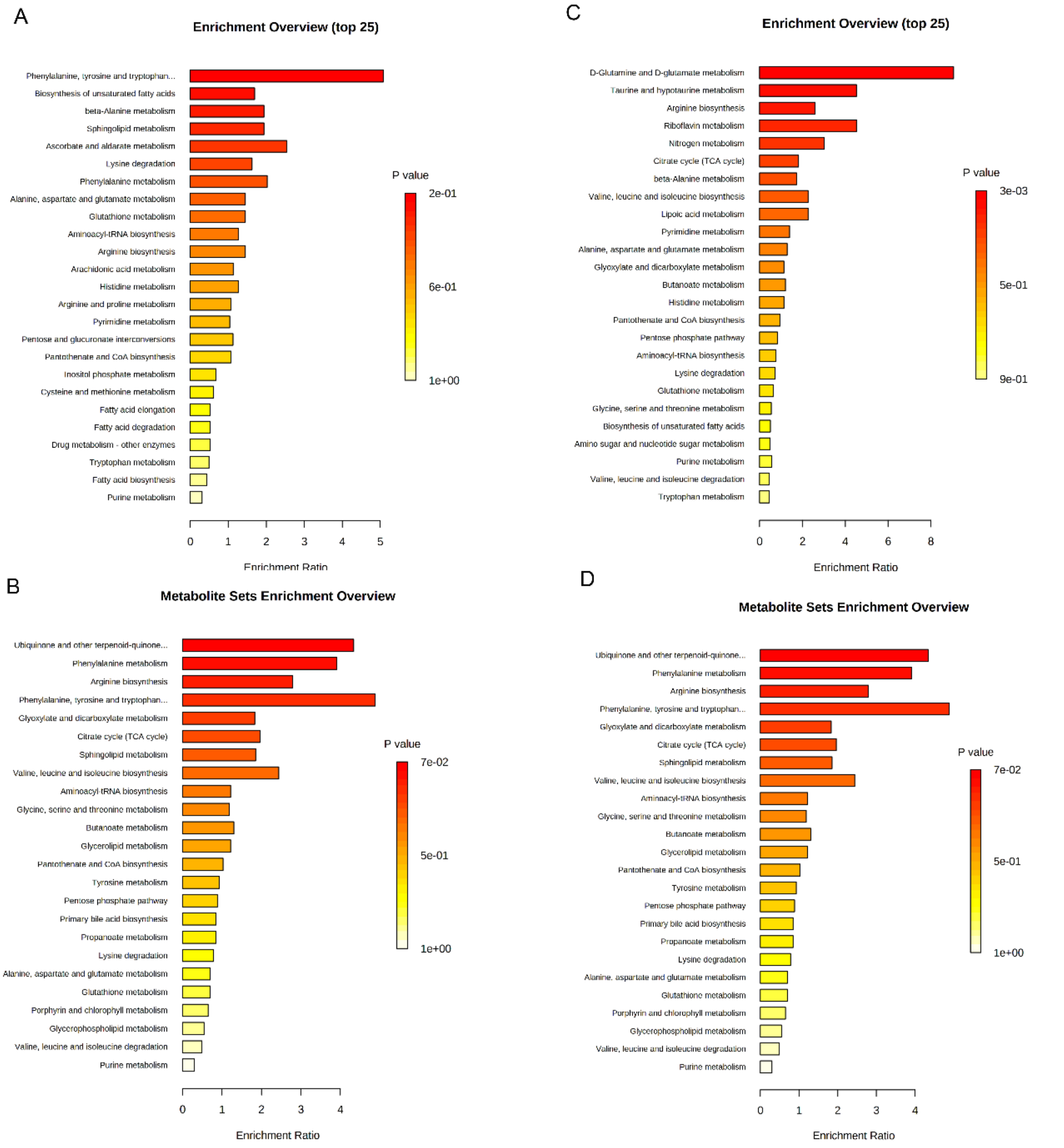

Supplementary Figure S1. Visualization of KEGG pathway enrichment analysis of all metabolites within the four modules. (A-D) KEGG analysis of all metabolites within the blue, brown, turquoise and yellow module.

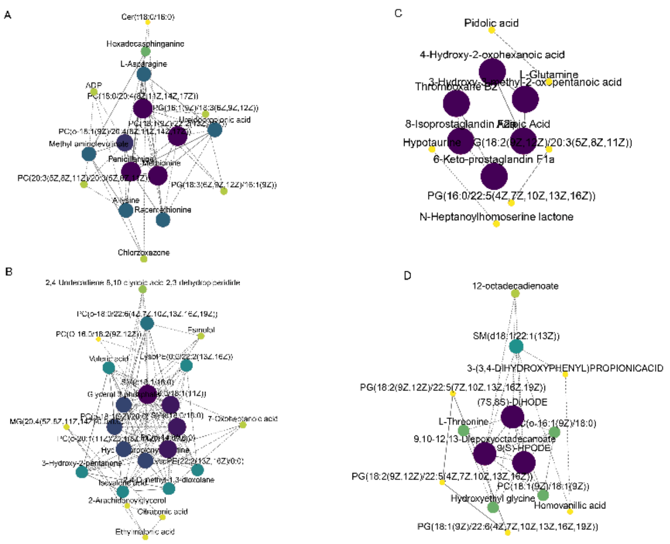

Supplementary Figure S2. Visualization of PPI network analysis of metabolites. (A-D) PPI network analysis of metabolites within the blue, brown, turquoise and yellow module. The darker the color of the dots, the higher the connectivity of the metabolites.

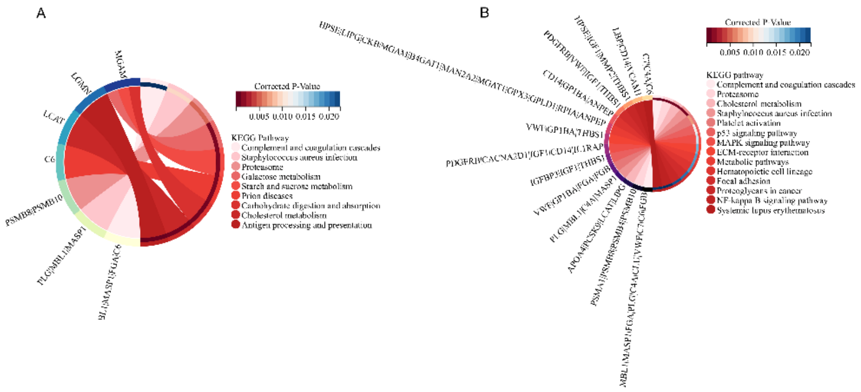

Supplementary Figure S3. Visualization of KEGG for proteins significantly correlated with four cytokine or four metabolite expression. (A) KEGG enrichment analysis of 30 key proteins in the protein - cytokine network; (B) KEGG analysis of 97 proteins from the protein-cytokines network. P value ≤ 0.05 were considered significant.

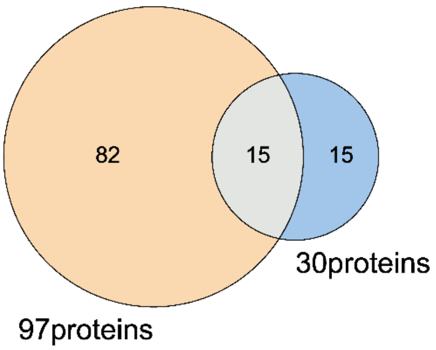

Supplementary Figure S4. Venn diagram of the 30 proteins in cytokines-metabolite and 97 proteins in protein-metabolite interaction network.
